# Prediction of nitrate and sulphate dynamics in groundwater under spatiotemporal effects of urban growth using attention optimized models

**DOI:** 10.1038/s41598-025-23423-y

**Published:** 2025-11-13

**Authors:** Dinesh Kumar Selvarangam, S. Jayalakshmi, S. S. Ramakrishnan

**Affiliations:** 1 Research Scholar, Institute of Remote Sensing, Anna University, Chennai, India; 2 Professor, Institute of Remote Sensing, Anna University, Chennai, India; 3 Former Professor, Institute of Remote Sensing, Anna University, Chennai, India

**Keywords:** Urban sprawl, Groundwater, Chengalpattu, Nitrate, Sulphate, Pollution, Environmental sciences, Hydrology

## Abstract

Groundwater quality in urban region is increasingly at risk due to the combined effects of urban sprawl, microclimatic conditions, and large sewage generation. Domestic Reverse Osmosis (RO) systems are unable to remove nitrate and sulphate in drinking water, and leads to human health hazards. This study focuses on prediction of nitrate and sulphate levels in groundwater through integrating microclimatic conditions with urban expansion indicators. A hybrid modeling approach has been developed using an Attention-based Convolutional Neural Network (ACNN) and Bayesian Optimized Multiple Linear Regression (BO-MLR). Sentinel satellite image is used for extraction of spectral band features, with attention scores highlights the most relevant indices for groundwater contamination. The above features have been combined with field-based measurements from sprawl-affected areas in the Chengalpattu region. To refine the dataset, the FP-Growth algorithm has been applied to identify strong associations between sprawl indicators and contaminant concentrations. The BO-MLR model has achieved prediction 95% of accuracy in detection of Nitrate and Sulphate levels in drinking water, closely match to the laboratory observations. Results shows that groundwater nitrate and sulphate level increases significantly with increase in urban sprawl, with 50% increase in built-up area linked to approximately 75% higher nitrate and 60% higher sulphate levels in groundwater. The above findings highlight the urgent need for sustainable urban planning and groundwater management strategies, provides awareness and hazardous zones in Chengalpattu area.

## Introduction

Groundwater contamination due to nitrates has emerged as a critical environmental hazards and leads to chronic health risk, especially in rapidly urbanizing regions. The expansion of urban sprawl are characterized through the proliferation of residential developments, roadways, and commercial zones, which disrupts the natural nitrogen cycle and contributes significantly to nitrate accumulation in groundwater^2,4,5^. The major primary sources of nitrate pollution is the leakage from septic systems, and deposits nitrogen-rich waste into the soil and eventually into aquifers^16,21^. Moreover, nitrate infiltration occurs through stormwater runoff, particularly in areas with impervious surfaces that expedite the transport of contaminants into the subsurface environment^1,14,28^. As a result, groundwater in peri-urban and urban–rural transition zones often exhibits elevated nitrate concentrations, frequently exceeding the safe drinking water thresholds set by public health guidelines^13,20^.

Urban sprawl alters land use and also affects the microclimatic conditions, which further exacerbate nitrate contamination. High-density built up environments contribute to the Urban Heat Island (UHI) effect, which increases soil temperatures and accelerates microbial processes, and converts organic nitrogen into more mobile nitrate forms^10–12^. The above condition enhances nitrate leaching and infiltration into groundwater systems. Moreover, urban sprawl leads to rapid runoff and increases recharge of contaminated water^3,6,7^. The above factors accumulates the nitrates in aquifers, leads to serious health risks and necessitates immediate intervention from urban planners and water resource managers^17,18,29^.

Similar trends are seen with sulphate contamination in groundwater, which is also exacerbated by urban sprawl and associated microclimatic changes. The expansion of impervious surfaces such as roads, buildings, and parking lots interferes with natural filtration processes, increases the potential for contamination from urban sources such as industrial effluents, sewage discharges, and stormwater runoff. The above sources are rich in sulphate compounds^9,13,19^. Poorly maintained wastewater infrastructure often results in the direct discharge of sulphate-laden effluents into groundwater systems^8,23^. Additionally, the widespread use of road salts, particularly in colder climates, induces compounds such as calcium sulphate into the environment, which are subsequently mobilized by stormwater runoff^25,27^. Increased rainfall intensity further accelerates this process, facilitates the entry of sulphate into groundwater^12,26^.

The combined effects of urbanization and microclimatic changes intensifes the frequency and magnitude of sulphate contamination in groundwater, prolonged drinking of water with sulphate leads to gastrointestinal distress and dehydration^22^. Urban infrastructure and environmental conditions such as elevated surface temperatures due to the UHI effect contribute to the chemical degradation of materials (i.e.) concrete and asphalt, which releases additional sulphate compounds into the subsurface^1,10^. Microclimatic changes, especially shifts in precipitation patterns and storm intensity, increases the likelihood of contaminant transport into aquifers^3,7^.

The simultaneous rise in nitrate and sulphate concentrations in groundwater due to urban sprawl lead to significant threat to human health. As cities expand, the movement of contaminants from sewage systems, fertilizers, and industrial discharges intensifies the sulphate and nitrate contamination in groundwater^5,13,19^. High nitrate levels in drinking water are linked to methemoglobinemia commonly referred as "blue baby syndrome" a condition that impairs oxygen transport in the blood and poses serious risks to infants^21,24^. Moreover, drinking water with nitrate leads to gastrointestinal disorders and cancers. Sulphate ingestion, at moderate level leads to diarrhoea and dehydration^22^. The compounding effects of urban-induced temperature alters the hydrological cycles and increases the mobilization and infiltration of these contaminants, exacerbates public health risks^6,10,15^. It is imperative to assess and monitor nitrate and sulphate levels in groundwater across urbanizing regions. Effective management strategies and stricter regulatory frameworks are essential to mitigate the contamination in groundwater and need to safeguard public health against these emerging environmental hazards.

### Problem statement

Urban sprawl, is associated with increased employment opportunities, economic investment, and infrastructure development, simultaneously contributes to significant environmental degradation particularly through air and water pollution. Environmental degradation is linked to urban expansion and rapidly increases the nitrate and sulphate concentrations in groundwater. Numerous studies have indicated that the spatial extent and intensity of urban sprawl are directly proportional to elevated levels of these contaminants in subsurface water resources.

The detection and assessment of nitrate and sulphate concentrations in relation to the directional growth of urban sprawl is a complex and underexplored challenge. Specifically, understanding how the geographic orientation and rate of urban expansion influence groundwater contamination patterns is critical for informed planning and mitigation efforts. Reverse Osmosis (RO) systems are commonly used in urban settings to treat drinking water, they partially remove nitrates and sulphates and typically eliminates only 80% of the ions. The remaining 20%, varies depending on membrane quality and filtration technology and still humans consume polluted water and lead to serious health risks. Furthermore, urban sprawl contributes to groundwater contamination through a range of pathways, such as increased agricultural runoff, stormwater infiltration, industrial discharge, inadequate wastewater management, loss of natural recharge zones, and alterations to local hydrological cycles. These impacts are compounded by microclimatic changes induced by urbanization, such as elevated temperatures and intensified precipitation events, which further exacerbate pollutant transport and infiltration into aquifers. The above multifaceted challenges need a integrated water resource management strategies that align with sustainable urban development objectives. A comprehensive understanding of the relationship between urban sprawl, microclimate variations, and groundwater contamination is essential. In particular, predictive models are capable of identifying the specific directions and extents of urban growth, which increases nitrate and sulphate levels in groundwater are identified. Such insights are vital for the design of targeted interventions, regulatory policies, and adaptive infrastructure planning that safeguard public health and ensures the long-term sustainability of urban water resources.

### Research questions

1. How does the dynamic expansion of urban sprawl affect the groundwater quality, particularly in terms of nitrate and sulphate level? Additionally, can regions within Chengalpattu characterized by high levels of urban sprawl but low levels of groundwater contamination can be identified, using microclimatic data as a determining factor?
2. What are the underlying microclimatic factors that contribute to low nitrate and sulphate levels in areas with significant urban sprawl? How does the directional pattern of sprawl expansion influence groundwater contamination, and what role does alter percolation and surface runoff play in waterbody pollution?
3. What is the relationship between urban sprawl, microclimatic variations, and groundwater contamination? Specifically, does urban expansion influence water percolation and contamination patterns in ways that are measurably linked to changes in local microclimate?

### Contributions

This study presents several key contributions toward understanding and addresses the relationship between urban sprawl, groundwater contamination, and microclimatic conditions, with a specific focus on the Chengalpattu region:

- To develop a novel framework which integrates deep learning and statistical models to examine the relationship between nitrate and sulphate concentrations in groundwater and urban sprawl. The proposed framework consists of an Attention-based Convolutional Neural Network (ACNN) for optimal spectral band selection and combination, TyDWT is used for image preprocessing, and PSO-DnCNN is used for perspective projection of water regions. The final prediction of contaminant levels is performed using Bayesian Optimized Multiple Linear Regression (BO-MLR) model, establishes robust connection between urban expansion and groundwater quality.
- To assess microclimatic influences on groundwater quality in urban sprawl regions through the proposed PSO-tuned DnCNN model, and various training algorithms, such as (i) *traingda* and (ii) *trainbr* are used. This enables accurate analysis of how local climatic shifts affect contaminant concentrations in urban environments.
- To Predict urban sprawl direction and spatial contamination mapping using spatial–temporal data collected for the Chengalpattu region. Proposed POA-DnCNN algorithm extracts image feature relevant to urban expansion, and then integrated with laboratory-verified water parameters specifically nitrate and sulphate concentrations for contamination levels prediction in areas undergoing rapid urbanization.
- To Predict water contamination due to microclimatic variation using the proposed BO-MLR model, provides actionable insights for the improvement of groundwater quality. This proposed framework supports for the sustainable urban planning and environmental management in rapidly growing urban regions.

## Material and methods

**Fig. 1:**
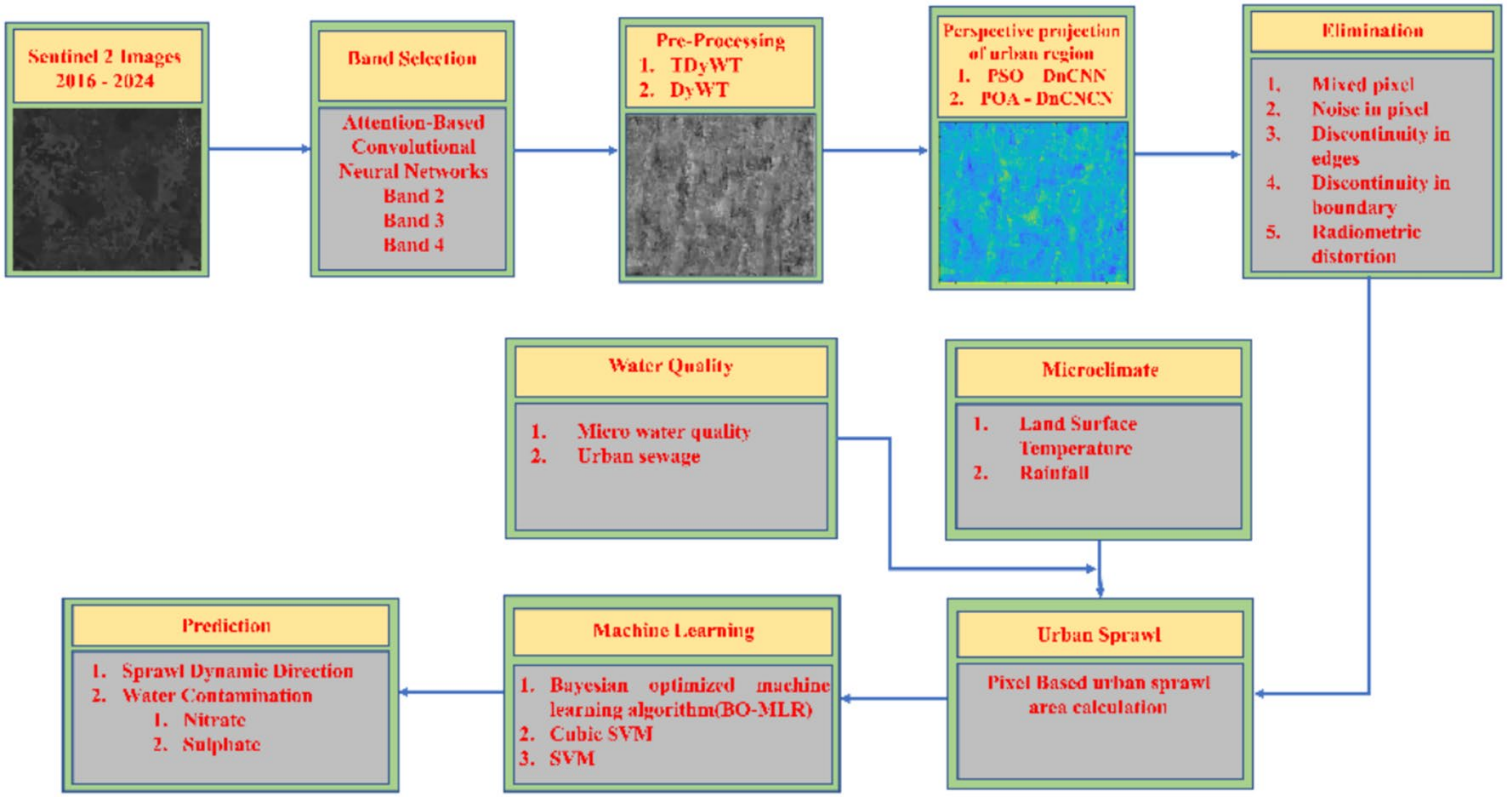
Overall methodology for prediction of relation between sprawl expansion and groundwater nitrate and sulphate levels.

The uncontrolled expansion of urban areas into rural land, impacts the ground water quality. The runoffs carries pollutant such as nitrates from fertilizers, sewage. Sulphates from industrial activities and road salts directly into rivers and lakes, degrades water quality and exhibits higher concentration of water pollutants. Urban sprawl affects ground water quality and disrupts natural habitats which has a major role in ecological balance. Urban sprawl significantly alters the local microclimates through interconnected environmental changes, with cascading effects on temperature, precipitation patterns, air quality, and ecological systems. This happens due to land-use transformations, which replaces the natural landscapes with impervious surfaces and dense infrastructure. The thermal absorption of concrete and asphalt store two to four times more heat than natural vegetation, urban temperatures is elevated compared to rural areas. Air conditioning and industrial activities in sprawled areas creates urban heat islands. Urban Heat Island (UHI) elevate heat-related mortality risks, particularly among vulnerable populations causing thermal stress which leads to change in microclimate. High-rise buildings fragment airflow, reduces wind speeds in dense urban areas and this stagnation exacerbates heat retention and pollutant accumulation. Sprawl increases short-term rainfall intensity by 20–30% due to enhanced convection from heat islands, which increases the chances flood risks with impermeable surfaces amplify runoff. The Fig. 1 shows the overall methodology for prediction of sprawl-based nitrate and sulphate level in groundwater.

### Study area and data collection

**Fig. 2:**
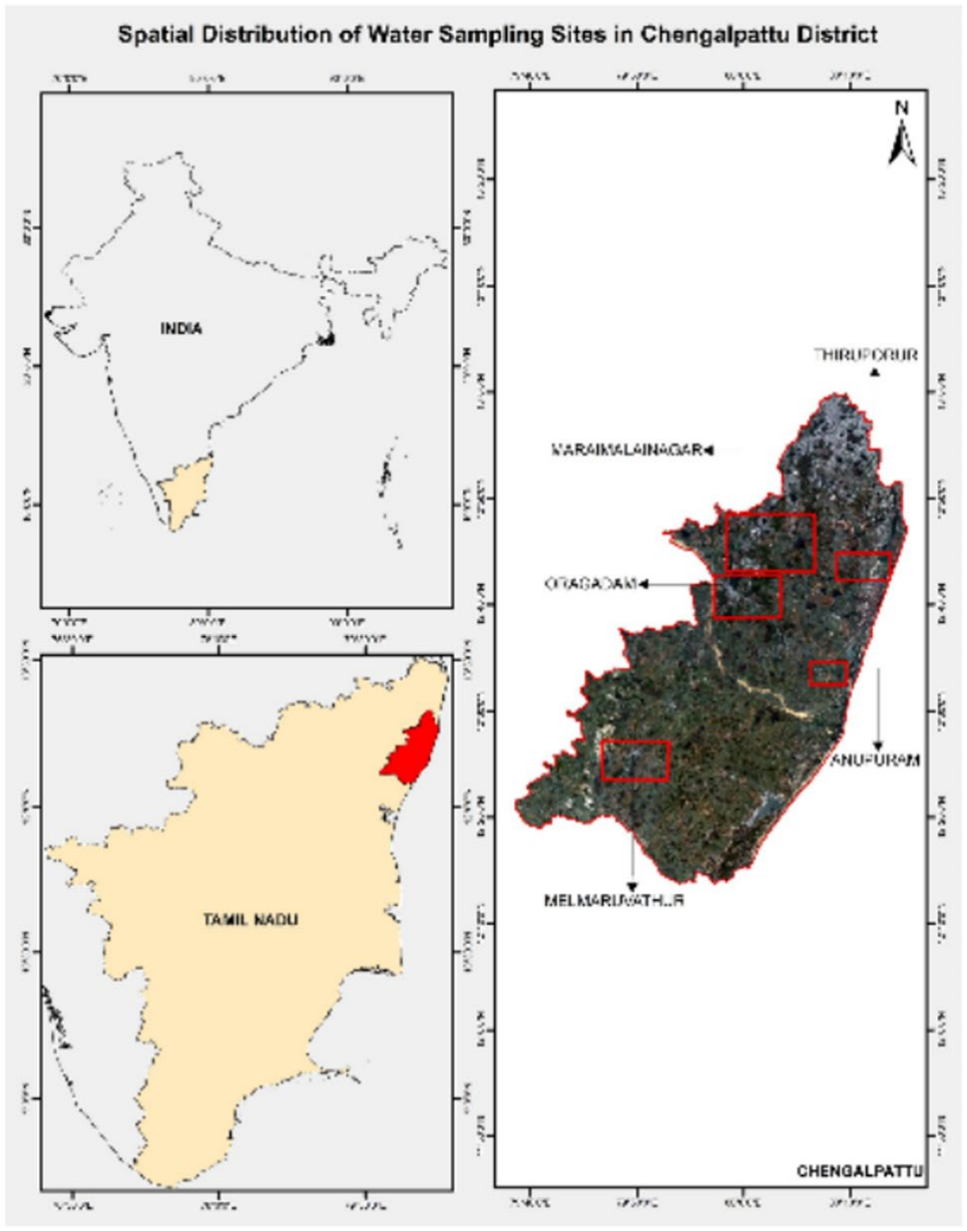
Study Area “Map prepared using ArcGIS Desktop 10.8 (Esri, https://www.esri.com)”.

Chengalpattu, is a developing town in Tamil Nadu, India, which has significant urban sprawl growth over the past two decades. Chengalpattu area has rapid industrial expansion, residential projects, and infrastructural growth and shown in Fig. 2. This urbanization has impact on groundwater contamination. Local aquifers in Chengalpattu has elevated nitrates level, heavy metals, and microbial contaminants. These changes not only threaten public health, and leads to long-term risks to the ecological balance of the area. Chengalpattu region need integrated urban planning and strict enforcement of environmental regulations to safeguard groundwater resources.

**Fig. 3:**
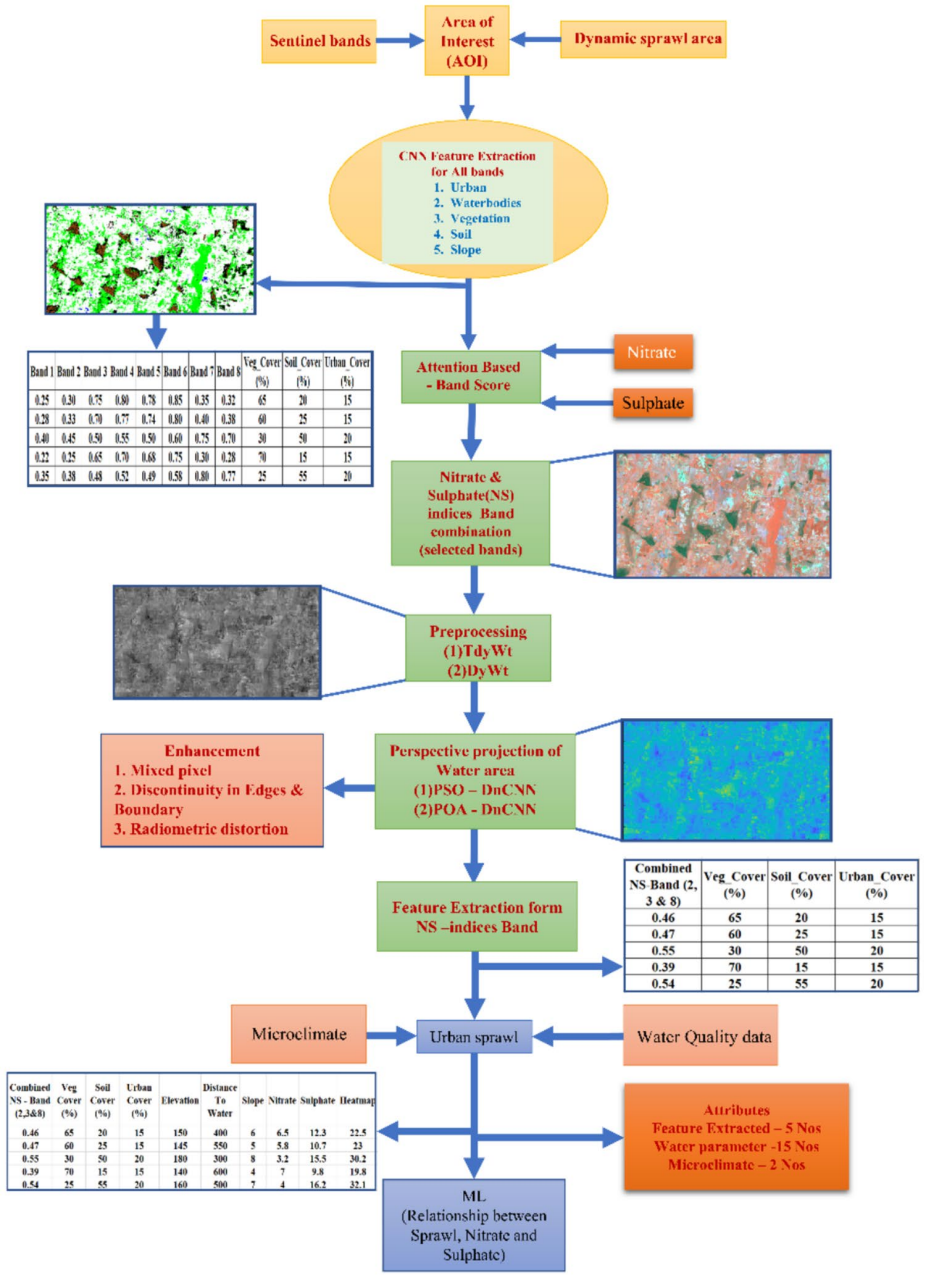
Conceptual diagram for extraction of NITRATE AND SULPHATE level in groundwater due to sprawl growth.

The nitrate and sulphate level in groundwater increases due to expansion of urban sprawl. For this study five areas (i)Thiruporur (ii)Oragadam (iii)Melmaruvathur (iv)Anupuram (v)Maraimalainagar are analysed for nitrate and sulphate levels and their relation with sprawl expansion. Figure 3 shows the conceptual diagram of the prediction of nitrate and sulphate levels due to sprawl growth.

## NS indices band combination using CNN algorithm

In this paper, Sentinel-2 satellite images are used for urban sprawl expansion measurement and its environmental impacts. Sentinel-2, has a spatial resolution ranging from 10 to 60 m across 13 spectral bands. The images are useful for tracking land use and land cover changes over time, allows to identify urban areas growth and encroachment upon natural landscapes. Sentinel-2 images are used for analysing vegetation health through indices such as NDVI and assessing soil moisture patterns, and detect the signs of contamination in groundwater. Additionally, the imagery helps in mapping potential pollution hotspots, such as industrial clusters and waste disposal sites, which are the contributors to nitrate and sulphate leaching into groundwater. When combined with field-based water quality data and geospatial modelling, Sentinel-2 imagery is used for spatial relationship between urban expansion and groundwater contamination, offers a powerful tool for environmental monitoring and urban planning.

### Data collection

In this paper, a dual approach was adopted for data collection to comprehensively assess the impacts of urban sprawl on groundwater contamination. Initially Sentinel-2 image was used to monitor land use changes and identify potential pollution sources across the study area. Through temporal analysis of high-resolution Sentinel-2 images, patterns of urban expansion such as industrial growth, and encroachment upon water bodies and green spaces were mapped. The selected band of remote sensing data is used for highlighting the areas of sprawl growth.

**Fig. 4:**
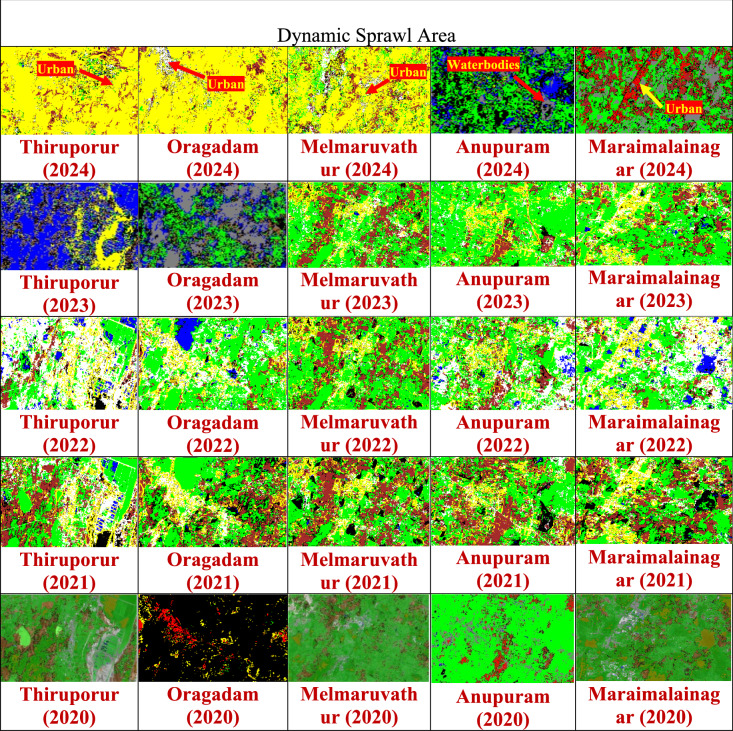
Spatial and temporal images to exhibit sprawl growth using CNN-algorithm.“Map prepared using MATLAB R2024a (MathWorks, https://www.mathworks.com).” Sentinel-2 data (Copernicus Open Access Hub, https://scihub.copernicus.eu/.

Further groundwater samples are collected from five areas such as (i)Thiruporur (ii)Oragadam (iii)Melmaruvathur (iv)Anupuram (v)Maraimalainagar. The samples were tested in the laboratory for key water contaminants and focused on nitrate and sulphate level concentrations, which are the indicators of pollution from sewage, and industrial discharge. By integrating satellite-based observations with direct water quality measurements, the study was able to draw meaningful correlations between land use dynamics and groundwater contamination. This method ensures accurate assessment of the environmental impacts of urban sprawl and pinpoint the critical zones, where nitrate and sulphate pollution levels are high. Figure 4 shows the Spatial and temporal images to exhibit sprawl growth using CNN-algorithm.

The satellite imagery used in Fig. 4 was obtained from Sentinel-2 MSI data accessed through the Copernicus Open Access Hub (https://browser.dataspace.copernicus.eu/). The acquisition details of the images are as follows: 2020 June 14, 2020, 05:15:19 (cloud cover: 6.2%); 2021 July 19, 2021, 04:57:01 (cloud cover: 3.8%); 2022 June 29, 2022, 05:15:32 (cloud cover: 1.5%); 2023 August 3, 2023, 04:56:59 (cloud cover: 1.4%); and 2024 September 16, 2024, 05:15:28 (cloud cover: 4.5%). The images were selected to ensure minimal cloud interference.

## Results

Sentinel-2 satellites provides rich multispectral data covering visible, near-infrared (NIR), and shortwave infrared (SWIR) wavelengths. Extracting meaningful features from this data is crucial for tasks such as land cover classification, vegetation monitoring, and urban mapping. Deep Convolutional Neural Networks (CNNs) are used for understanding spatial and spectral patterns directly from data. The different architectures are VGG, ResNet, GoogLeNet, EfficientNet/MobileNet/DenseNet, EfficientNet, MobileNet and DenseNet. Table 1 shows Different Architectures. Table 2 shows the CNN based sentinel image features using Efficient Net-B0.

**Table 1:** Different Architectures**.**

Model	Bands Used (Sentinel-2)	Num Bands	Overall Accuracy (%)	Precision (%)	Recall (%)	F1 Score (%)	Extraction Time (s/img)	Params (M)
VGG16	B2, B3, B4 (RGB)	3	84.2	82.7	83.5	83.1	0.85	138
VGG16	B4, B8, B11 (Red, NIR, SWIR)	3	86.3	84.9	85.7	85.3	0.86	138
ResNet-50	B2, B3, B4, B8	4	88.2	87.1	87.6	87.3	0.66	25.6
ResNet-50	B4, B5, B6, B7, B8A	5	89.4	88.1	89.0	88.5	0.65	25.6
GoogLeNet	B3, B4, B8, B11	4	87.1	86.3	86.8	86.5	0.58	6.8
DenseNet-121	B2–B4, B5–B8A, B11, B12	10	90.2	89.4	90.1	89.7	0.76	8.0
EfficientNet-B0	B2–B4, B5, B6, B8, B11	7	91.0	90.2	91.1	90.6	0.42	5.3

**Table 2:** CNN based sentinel image features using Efficient Net-B0.

BAND1	BAND2	BAND3	BAND4	BAND5	BAND6	BAND7	BAND8	VEG_COVER (%)	SOIL_COVER (%)	URBAN_COVER (%)
0.25	0.3	0.75	0.8	0.78	0.85	0.35	0.32	65	20	15
0.28	0.33	0.7	0.77	0.74	0.8	0.4	0.38	60	25	15
0.4	0.45	0.5	0.55	0.5	0.6	0.75	0.7	30	50	20
0.22	0.25	0.65	0.7	0.68	0.75	0.3	0.28	70	15	15
0.35	0.38	0.48	0.52	0.49	0.58	0.8	0.77	25	55	20

From Table 2 shows the feature extraction from Sentinel images. In Band each pixel in the image has a numeric value. This value is the reflectance or radiance. Sunlight is reflected by the Earth’s surface at that band’s wavelength i.e., 443 nm for Band 1. The range normally from 0 to 1 (sometimes scaled 0–100% or 0–10,000 in raw satellite data). In Band 1 value is 0.25, this means 25% of the sunlight at ~ 443 nm (blue light) is reflected by the surface at that pixel. The Vegetation Cover (%) is calculated based on the equation, Vegetation Cover (%) = ((NDVI—NDVI_soil)/(NDVI_veg—NDVI_soil)) × 100; Where: NDVI = Normalized Difference Vegetation Index value; NDVI_soil = NDVI value for bare soil (typically around 0.1); NDVI_veg = NDVI value for dense vegetation (typically around 0.8). Band score calculation of NS indices bands using Attention based band score prediction. Table 2 shows the band score for NS indices band.

### Attention score based cnn for band selection: NS indices band

Attention scores quantify the importance of each spectral band or groups of bands in a classification task like land cover mapping. Table 3 shows the Attention based band Score predictions for NS indices.

**Table 3:** Attention based band Score predictions for NS indices.

3-BAND COMBINATION	TOTAL ATTENTION SCORE
Band 2 (Green), Band 3 (Red), Band 4 (NIR)	0.55
Band 3 (Red), Band 4 (NIR), Band 8 (Narrow NIR)	0.54
Band 3 (Red), Band 4 (NIR), Band 7 (Red Edge)	0.52
Band 1 (Blue), Band 3 (Red), Band 4 (NIR)	0.52
Band 2 (Green), Band 4 (NIR), Band 8 (Narrow NIR)	0.51
Band 3 (Red), Band 4 (NIR), Band 5 (SWIR1)	0.5
Band 2 (Green), Band 4 (NIR), Band 7 (Red Edge)	0.49
Band 1 (Blue), Band 2 (Green), Band 4 (NIR)	0.49
Band 4 (NIR), Band 7 (Red Edge), Band 8 (Narrow NIR)	0.48
Band 1 (Blue), Band 4 (NIR), Band 8 (Narrow NIR)	0.48

The typical process involves these steps:

**Step 1:** Extract Features from Each Band.

Each spectral band of the input image is processed through convolutional layers, either independently or jointly. This produces feature maps that capture both spatial and spectral information relevant to that band.

**Step 2:** Compute Raw Importance Scores.

An attention module often a small neural network is applied to these feature maps to generate a raw importance score for each band. This usually involves (i) Applying global average pooling to condense spatial features into a vector for each band. (ii) Passing these vectors through fully connected layers and obtained raw scores that represent how relevant each band is to the task.

**Step 3:** Normalize Scores to Get Attention Weights.

The raw scores are converted into normalized attention weights using a function like the softmax. This ensures all weights are positive and sum to one across the bands:

αᵢ = e^(sᵢ)/∑(e^(sⱼ)) for j = 1 to N, where sᵢ is the raw score of the iᵗʰ band, and αᵢ is its normalized attention weight.

**Step 4:** Calculate Scores for Band Combinations.

To evaluate a combination of bands (for example, bands 2, 3, and 4), the model sums their individual attention weights:

Total Attention Score_combo = ∑ αᵢ for all i in the combo.

This sum reflects the overall importance of the selected band set in the model’s decision-making. By learning to assign higher attention weights to more informative bands, the model effectively selects spectral bands that contribute for accurate classification, improves efficiency and interpretability.

### Pre-process of NS indices band for sprawl region enhancement

**Fig. 5:**
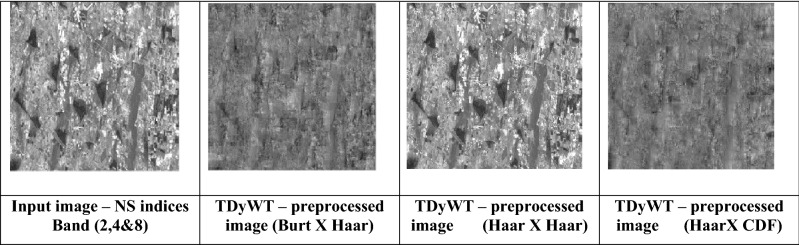
TDyWt Enhanced regions of sprawl regions.

In this paper, Transfer Dyadic Wavelet Transform (TDyWt) and Dyadic Wavelet Transform (DyWt) are used for pre-processing and enhances the sprawl region. The wavelet breaks the satellite images into different frequency components, enhances the satellite image for better extraction of sprawl features. TDyWt enhances spatial variations at multiple scales and sharpens the sprawl patterns and enhances land cover region. DyWt reduces a noise and enhances surface features such as vegetation health and soil moisture, which are indirect indicators of groundwater quality. The TDyWt enhances the industrial regions with visual and spatial accuracy. Figure 5 shows the enhanced images using TDyWt. Table 4 shows the statistical metric of TDyWt and DyWt. TDyWt performs better than DyWt due to dyadic scaling.

**Table 4:** Statistical Metrics for Each Pre-processed Image.

Image Type	Mean Intensity	Standard Deviation	Entropy	Variance	PSNR (dB)	SNR (dB)
Input (Bands 2,4,8)	0.45	0.18	6.80	0.032	N/A	N/A
TDvDWT – Decomposition Haar wavelet × Reconstruction Haar wavelet	0.43	0.22	7.10	0.048	28.5	24.3
TDvDWT—Burt × Haar wavelet	0.48	0.20	7.25	0.040	29.2	25.1
TDvDWT—Haar wavelet × CDF	0.40	0.15	6.55	0.022	27.8	23.5

Entropy: Measures the information richness. Higher entropy means more details. PSNR is the Peak Signal-to-Noise Ratio. Higher PSNR proves the better reconstruction quality (closer to the original). SNR is the Signal-to-Noise Ratio. Higher SNR means cleaner image with less noise. The TDvDWT with Burt × Haar during decomposition reconstruction provide the highest entropy (7.25), highest PSNR (29.2 dB) and highest SNR (25.1 dB). TDyWt enhanced image is processed with DnCNN for perspective projection of sprawl region.

### Perspective projection of sprawl region using PSO-DnCNN & POA-DnCNN algorithm

**Fig. 6:**
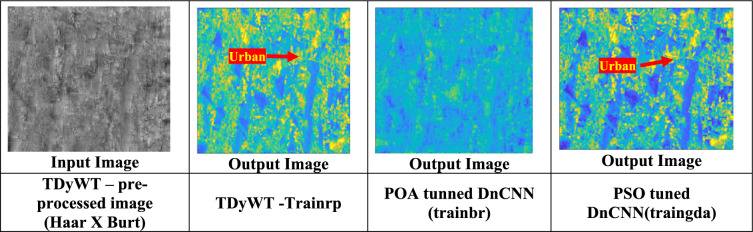
TyDyWt processed input image to DnCNN and output image of DnCNN.

In this paper, perspective projection of sprawl region is performed using PSO-DnCNN (Particle Swarm Optimization-based Denoising Convolutional Neural Network) and POA-DnCNN (Penguin Optimization Algorithm-based DnCNN). The proposed POA-DnCNN and PSO-DnCNN methods sharpen the sprawl region, which are smooth in nature due to atmospheric disturbances. PSO-DnCNN uses the principles of particle swarm optimization to fine-tune the neural network parameters and prespectively project the sprawl regions. POA-DnCNN achieves the similar goal, enhances the model’s robustness and efficiency. The perspective projected images have provided clear insights of urban growth patterns, industrial clusters, and environmental degradation, which are critical for identification of potential sources of groundwater pollution. The sharper sprawl regions which are perspective projected used for precise spatial correlation between urban sprawl and groundwater contamination management. Figure 6 shows the TDyWt input image and proposed perspective projection images.

**Fig. 7:**
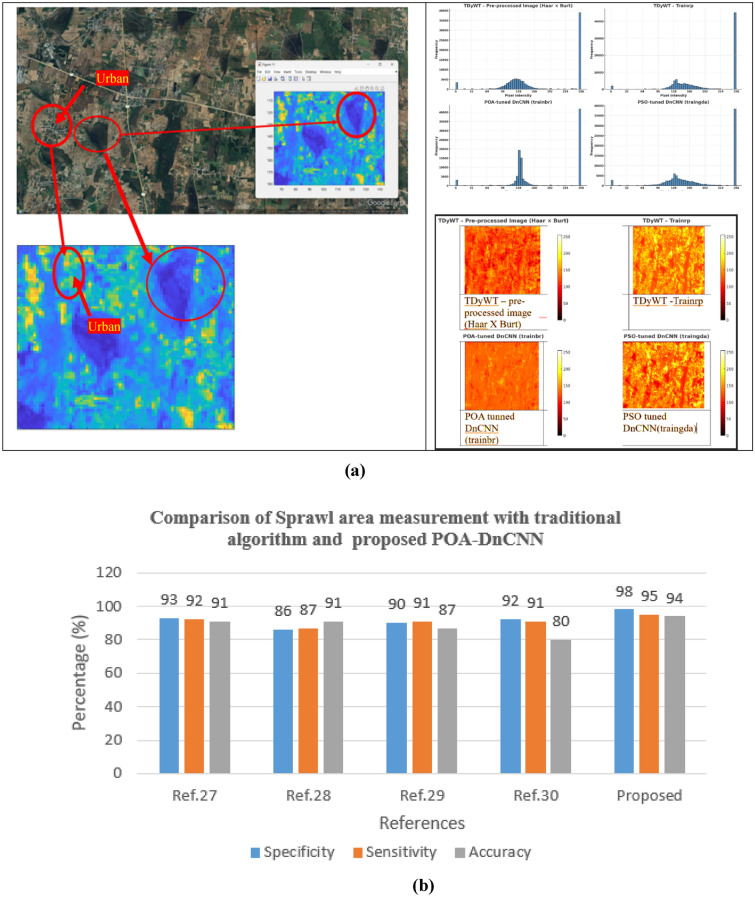
(**a**) Heat map of POA tunned DnCNN with different training functions “Map prepared using MATLAB R2024a (MathWorks, https://www.mathworks.com).” (**b**) Comparison of Sprawl area measurement with traditional algorithm and proposed POA-DnCNN.

Table 5 shows the statistical analysis of proposed POA-DnCNN, PSO-DnCNN algorithms with different training functions such as trainrp, trainbr and traingda. Figure 7(a) showsHeat map of POA tunned DnCNN with different training functions. Figure 7(b) Comparison of Sprawl area measurement with traditional algorithm and proposed POA-DnCNN. Table 6 shows the POA and PSO tunned DnCNN algorithms performance in contrast and structured similarly indices.

**Table 5:** Compare the proposed POA and PSO tunned DnCNN algorithm.

Image Type	Entropy	PSNR (dB)	SNR (dB)	Remarks
TDyWT – preprocessed (Haar × Burt)	~ 6.9	~ 28.0	~ 24.0	Base pre-processed, smooth detail, good for further processing
TDyWT—Trainrp	~ 7.4	~ 29.5	~ 25.5	Better texture extraction, moderate noise
POA-tuned DnCNN (trainbr)	~ 7.8	~ 30.8	~ 26.8	Strong denoising, sharp features, best balance of quality
PSO-tuned DnCNN (Traingda)	~ 7.2	~ 29.0	~ 25.0	Balanced result, slightly noisier than DnCNN

**Table 6:** Compare the POA and PSO tunned DnCNN algorithms for contrast and structured similarly indices.

Image	Mean	Std Dev	Entropy	PSNR (dB)	SNR (dB)	SSIM
TDyWT – Pre-processed Image (Haar × Burt)	170.93	72.58	3.37	∞	∞	1.00
POS-DnCNN (Trainrp)	192.61	62.16	3.18	11.94	−0.98	0.32
POA-tuned DnCNN (trainbr)	178.54	68.70	2.65	12.58	−3.70	0.37
PSO-tuned DnCNN (traingda)	179.83	66.49	3.57	11.99	−0.58	0.34

### Linking sprawl growth and microclimate to groundwater degradation

**Fig. 8:**
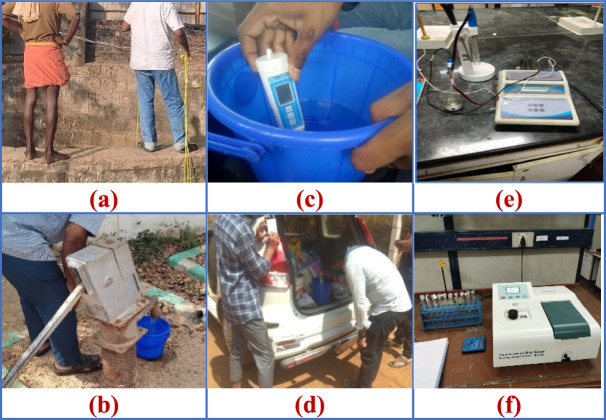
(**a**) Open well (**b**) Bore well (**c & d**) Onsite checks (**e & f**) Lab Testing.

The water samples are collected from Chengalpattu district from the coastline to inland and analyse the diversity of water conditions across the region. Satellite imagery and GIS tools have been used in the study to identify and map various features such as water bodies, urban areas, agricultural zones, and industrial areas. By layering this information, sampling points were carefully selected based on varying environmental and human influences on water quality throughout the district. Samples have been collected from different sources such as (a) open wells, (b) bore wells, and surface water bodies such as ponds and tanks. To maintain consistency and aid in future referencing, GPS coordinates were recorded for each location. In addition to collecting samples for lab testing, quick on-site checks were done using Lutron and Eutech handheld meters. These meters provided real-time measurements of parameters such as dissolved oxygen (DO) and total dissolved solids (TDS), which are useful indicators of water health and possible pollution. Figure 8 shows the photograph of sample collection. Table 7 shows the Water quality parameters of Chengalpattu district.

**Table 7:** Water quality parameters of Chengalpattu district.

Location	pH	EC (uS/cm)	Total Hardness (mg/l)	Calcium	Magnesium (mg/l)	TDS (mg/l)	CHLORIDE (mg/l)	SALINITY (PSU)	SODIUM (mg/l)	POTASSIUM (mg/l)	NITRATE (mg/l)	SULPHATE (mg/l)	FLUORIDE (mg/l)	TP (mg/l)	Total Alkalinity (mg/l)	Veg Cover	Soil Cover	Urban Cover	Temp	Rainfall
1	7.80	3412.50	1149.53	422.00	199.16	2547.24	1203.04	4.32	674.76	26.76	82.13	452.15	5.93	4.13	805.10	32.47	25.52	58.27	32.84	251.86
2	9.11	6610.21	1041.53	345.44	88.86	2304.14	1122.23	5.48	273.54	31.14	87.75	722.26	2.45	2.21	383.86	67.04	40.33	32.94	31.41	274.02
3	7.06	5021.01	1487.70	298.48	146.46	1964.29	910.11	4.43	663.29	27.08	104.85	752.10	3.59	3.35	777.16	53.92	13.99	87.87	25.38	129.5
4	9.04	6305.83	950.92	467.22	196.32	2720.69	584.56	4.46	446.06	23.55	62.20	847.26	5.88	2.54	420.98	45.92	28.14	74.26	26.62	77.51
5	7.70	4280.20	717.07	188.92	142.77	2527.10	1797.08	5.57	329.12	24.83	75.15	659.00	5.46	2.25	398.19	19.36	31.66	85.76	25.47	106.98
6	7.30	6582.09	742.99	322.42	155.53	1743.93	1719.35	3.91	429.57	38.43	102.80	476.55	5.27	5.96	398.56	19.36	7.09	82.64	34.55	156.78
7	8.74	4556.81	1361.51	153.97	163.49	2866.39	1999.58	2.97	690.02	28.25	113.71	570.27	3.03	3.29	788.74	13.49	32.34	61.85	29.72	254.5
8	8.57	3043.35	1218.22	314.03	134.54	2733.81	1994.96	2.60	446.31	34.57	90.70	617.85	2.68	5.24	699.12	61.97	12.67	84.53	32.63	265.18
9	9.19	6621.53	701.12	169.71	155.31	2924.70	1333.15	5.14	364.38	40.14	91.93	787.55	4.67	3.02	613.84	46.07	7.93	26.19	38.61	51.74
10	8.84	3365.15	684.11	191.59	150.12	2588.58	1653.48	4.67	516.70	47.62	66.43	742.47	5.72	4.73	515.30	52.48	47.7	33.72	28.74	177.69
11	9.01	4277.25	1300.97	191.13	188.14	2420.12	1917.15	4.96	320.07	21.57	86.84	613.66	4.23	5.04	826.32	11.24	48.45	23.17	31.16	154.35
12	7.71	6800.25	672.76	377.22	85.45	2127.36	1774.47	3.25	237.93	51.98	81.96	991.91	4.29	4.38	535.47	68.19	41.38	42.77	36.33	105.53
13	7.44	6802.43	1421.86	411.12	113.72	2899.09	871.02	2.98	264.44	45.12	54.55	763.46	3.12	3.89	789.96	59.95	18.71	47.21	28.43	79.97
14	8.88	5293.75	1306.24	354.18	194.05	2799.10	1175.82	2.55	264.02	23.27	76.15	542.34	5.08	3.65	563.48	22.74	9.4	38.99	26.15	134.4
15	9.02	5527.35	681.35	486.76	186.83	1567.83	693.74	3.73	275.95	54.94	82.64	461.07	2.75	3.40	526.17	20.91	35.79	78.01	29.35	285.73
16	9.48	4793.78	684.84	281.20	134.68	1539.55	1931.08	4.56	269.41	56.83	81.20	491.72	3.29	5.72	577.61	21	24.81	44.97	27.42	130.8
17	8.03	4172.84	1586.64	250.00	154.42	2064.70	1409.26	3.87	520.44	22.44	79.32	547.57	3.70	5.32	480.83	28.25	10.49	39.67	38.95	179.7
18	7.93	4314.66	974.27	454.01	113.29	2715.83	842.96	4.03	290.94	31.08	42.69	496.41	4.03	5.86	748.57	41.49	27.28	57.99	37.12	225.75
19	8.94	5690.07	970.64	228.26	102.57	2980.91	1507.55	5.66	372.83	52.25	79.65	511.94	2.97	2.50	601.63	35.92	6.55	29.86	34.5	140.91
20	7.85	6009.50	1412.80	487.13	135.64	1725.63	1427.19	3.72	648.39	49.93	87.19	571.06	2.46	4.92	439.33	27.47	45.92	76.15	38.07	292.95
21	9.33	6166.32	1547.25	154.25	122.40	2391.20	1037.24	4.30	436.98	27.38	113.43	504.02	4.44	5.75	839.74	46.71	16.65	25.22	37.06	290.61
22	9.15	6158.47	1586.00	489.46	150.04	2071.34	670.34	5.24	533.78	28.37	95.62	938.06	3.15	2.72	530.33	18.37	34.81	89.08	27.8	112.95
23	8.07	3364.82	1353.38	165.11	89.33	2954.87	1507.36	3.89	286.16	34.82	100.75	448.14	4.32	2.27	626.13	27.53	19.03	74.06	38.39	174.31

### Association rule-based BO-MLR for prediction of sulphate and nitrate level in sprawl regions

Groundwater quality is based on land use, temperature (microclimate), and hydrological processes that vary spatially and temporally. Water quality data and microclimatic data from ground measurements, and sprawl data are obtained from satellite image after pre-processed with TyDWt and POA-DnCNN image. Integrating these heterogeneous data for varying spatial resolutions, temporal frequencies are used for predicting the relation between sprawl and Nitrate, Sulphate in groundwater. For accurately identifying the relation between the inputs requires spatial data, microclimatic data and water quality parameters. Integrating water quality, microclimatic, and urban sprawl data for predictive modelling have challenges related to data heterogeneity, complex interdependencies, limited ground truth, dynamic urbanization impacts, and modelling complexity.

In this paper, association rule mining-based FP-Growth (Frequent Pattern-Growth) is proposed and improves the prediction of sulphate levels in urban areas. Uncovers the hidden and meaningful relationships among multiple environmental and urbanization-related variables. The proposed method FP Growth method enhances sulphate level prediction. Association rule mining-based FP-Growth algorithm identifies frequent patterns and correlations between sulphate concentrations and influencing factors such as water quality parameters, microclimatic variables such as temperature, humidity, precipitation, and urban sprawl indicators such as impervious surfaces, land use changes. In the FP-Growth method, Feature Selection and Dimensionality Reduction, extracting strong association rules with high confidence and support, the method highlights the relevant variables and their interactions which are associated with sulphate contamination. The feature set improves the performance and interpretability of predictive models such as multiple linear regression (MLR), reduces the noise and overfitting.

Association rule-based FP-Growth algorithm extracted features are applied with Bayesian Optimization of MLR parameters (BO-MLR), for analysis of nonlinearities and interactions specific to urban sprawl contexts. Bayesian optimized multilevel regression (BO-MLR) algorithm combines the hierarchical modelling capabilities of multilevel (or hierarchical) regression with the efficient parameter tuning and uncertainty quantification provided by Bayesian optimization techniques. Multilevel Regression Models is known as hierarchical linear models, allow for data structured at multiple levels. They estimate parameters at each level, acquires both within-group and between-group variability. Bayesian multilevel models incorporate prior information and provides full posterior distribution for parameter, improves inference especially when data are sparse or groups are few.BO is based on Gaussian Process as objective function and iteratively selects promising points hyperparameter tuning in to handle noisy, expensive evaluations.BO-MLR optimizes hyperparameters such as variance components, regularization parameters, or model complexity settings and improves predictive performance. BO efficiently searches the hyperparameter space, often outperform the traditional methods.

### Sulphate level prediction using bo-mlr

**Table:**
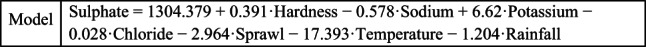

In the above equation, dependent variable is Sulphate concentration, which is the linear function of independent environmental and chemical variables.

This model attempts to quantify the influence of various environmental and chemical parameters on sulphate levels. The intercept of 1304.379 suggests a baseline sulphate concentration in the absence of all other predictors. Each coefficient represents the expected change in sulphate concentration per unit change in the respective variable, holding all other factors constant. For instance, for every unit increase in water hardness, the sulphate concentration is increased approximately 0.391 units. Similarly, potassium shows a strong positive association with sulphate (6.62), while temperature has a substantial negative effect (−17.393), indicates that higher temperature reduces sulphate levels. Overall, the model serves as a valuable analytical tool for understanding the multifactorial influences on sulphate levels in environmental or water quality studies.

The Table 8 shows BO-MLR model sulphate level concentration prediction based on a range of environmental and chemical variables.

**Table 8:** Summary of BO-MLR for FP-Growth feature dataset for Sulphate prediction.

Predictor	Coefficient	Estimate	Standard Error	t-statistic	p-value
Constant	β₀	1304.379	338.831	3.85	0.061
Hardness	β₁	0.391	0.183	2.133	0.167
Sodium	β₂	−0.578	0.260	−2.225	0.156
Potassium	β₃	6.62	5.546	1.194	0.355
Chloride	β₄	−0.028	0.082	−0.336	0.769
Sprawl	β₅	−2.964	1.679	−1.766	0.220
Temperature	β₆	−17.393	8.065	−2.156	0.164
Rainfall	β₇	−1.204	0.501	−2.402	0.138

The predictors include hardness, sodium, potassium, chloride, urban sprawl, temperature, and rainfall. Each row provides the estimated coefficient for a variable, along with the standard error, *t*-statistic, and *p*-value, offering insight into both the direction and statistical strength of each relationship.

The overall model fit is presented in the Table 9 provides key insights into how well the regression model explains variations in sulphate concentration. The R-squared value of 0.913 suggests that approximately 91.3% of the variability in sulphate levels.

**Table 9:** Summary of Overall Fit.

Summary of Overall Fit	
R-Squared	0.913
Adjusted R-Squared	0.608
Residual Standard Error	82.74 on 2 degrees of freedom
Overall F-statistic	2.993 on 7 and 2 degrees of freedom
Overall p-value	0.273

The ANOVA Table 10 shows the statistical breakdown of the variance in sulphate concentrations and explains the BO-MLR model. This analysis helps assess the overall strength of the model by decomposing the total variability in the outcome variable into components attributed to the model and residual (unexplained) error. The regression sum of squares (SS) is 143,412.681, which represents the portion of the total variance in sulphate levels that is explained by the set of predictor variables.

**Table 10:** Analysis of Variance.

Analysis of Variance (ANOVA)					
**Source**	**df**	**SS**	**MS**	**F-statistic**	**p-value**
Regression	7	143,412.681	20,487.526	2.993	0.273
Residual Error	2	13,691.862	6,845.931		
Total	9	157,104.544	17,456.06		

The mean square (MS) values are derived by dividing each sum of squares by its respective degrees of freedom. The mean square for regression is 20,487.526, while the mean square for the residual error is 6,845.931. These values are used to compute the F-statistic**,** which in this case is 2.993. This statistic tests explains the variation in sulphate levels.

**Fig. 9:**
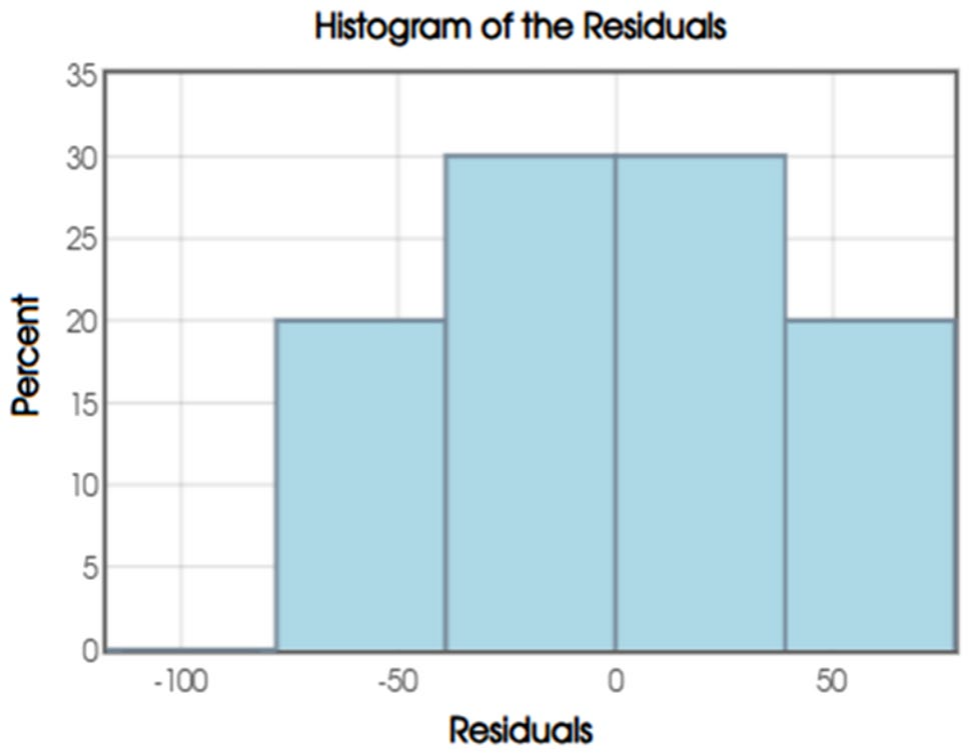
Histogram of the Residuals.

**Fig. 10:**
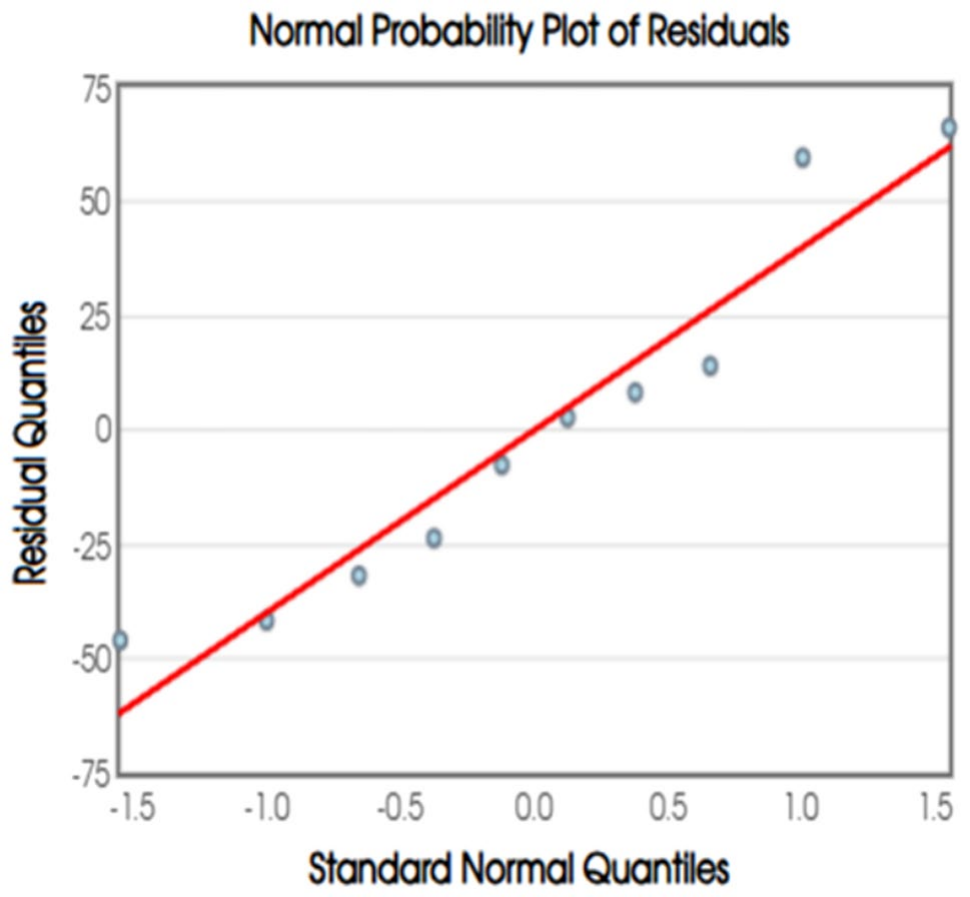
Normal Probability Plot of Residuals.

The histogram of the residuals shows the visual assessment of the error distribution in the BO-MLR model as in Fig. 9 and predicted sulphate concentration. Residuals represent the differences between observed and predicted values. The normal probability plot (or Q-Q plot) shown in Fig. 10 does a diagnostic check on whether the residuals from the regression model follow a normal distribution which is the key assumptions underpinning BO-MLR analysis.

The descriptive statistics Table 11 summarizes the distribution of residuals from the regression model, offers a quantitative lens through which to assess the spread and central tendency of the prediction errors.

**Table 11:** Five Number Summary of Residuals.

Statistic	Value
Minimum	−45.987
1 st Quartile (Q₁)	−31.841
Median (M)	−2.457
3rd Quartile (Q₃)	14.041
Maximum	66.179

### Nitrate level prediction using Bo-Mlr

**Table:**
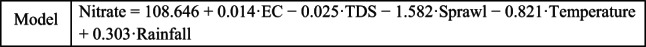

The BO-MLR model presented in the above equation describes the relationship between nitrate concentration and a set of environmental predictors: electrical conductivity (EC), total dissolved solids (TDS), sprawl, temperature, and rainfall. The BO-MLR equation shows a simplified snapshot of multiple environmental, anthropogenic factors which influences the nitrate concentration. Model’s effectiveness depends on further diagnostics such as significance tests, residual analysis. The analysis provides a foundation for understanding and predicting nitrate variability across landscapes.

The Table 12 shows BO-MLR coefficients and statistical significance of five predictors used to model nitrate concentrations: electrical conductivity (EC), total dissolved solids (TDS), urban sprawl, temperature, and rainfall. Each predictor’s estimate is accompanied by its standard error, t-statistic, and p-value. The intercept (β₀ = 108.646) represents the baseline nitrate level when all other variables are set to zero. This estimate is statistically significant (p = 0.006), suggesting a meaningful baseline concentration in the absence of other contributing factors. Predictors, EC (β₁ = 0.014, p = 0.009) and rainfall (β₅ = 0.303, p = 0.009) both show statistically significant positive associations with nitrate levels. This suggests that increases in water conductivity and precipitation are likely to correspond with elevated nitrate concentrations, possibly due to runoff or leaching from surrounding environments. Table 12 provides evidence that EC, TDS, sprawl, and rainfall significantly contribute to nitrate variability, with EC and rainfall increasing nitrate presence.

**Table 12:** Regression Analysis of BO-MLR.

Predictor	Coefficient	Estimate	Standard Error	t-statistic	p-value
Constant	β₀	108.646	20.285	5.356	0.006
EC	β₁	0.014	0.003	4.764	0.009
TDS	β₂	−0.025	0.004	−5.983	0.004
Sprawl	β₃	−1.582	0.256	−6.187	0.003
Temperature	β₄	−0.821	0.441	−1.860	0.136
Rainfall	β₅	0.303	0.064	4.763	0.009

The Table 13 Summary of Overall Fit and the performance, reliability of the regression model used in the study. With an R-squared value of 0.936**,** the model explains approximately 93.6% of the variability in the dependent variable, indicates a very strong fit.

**Table 13:** Summary of Overall Fit.

Summary of Overall Fit	
R-Squared	0.936
Adjusted R-Squared	0.856
Residual Standard Error	5.573 on 4 degrees of freedom
Overall F-statistic	11.675 on 5 and 4 degrees of freedom
Overall p-value	0.017

To evaluate the overall significance of the regression model, an ANOVA test was conducted. The results are presented in Table 14. The analysis shows that the BO-MLR model accounted for a substantial portion of the total variability in the response variable, as indicated by the sum of squares for regression (SS = 1813.06) compared to the residual error (SS = 124.24).

**Table 14:** Analysis of Variance.

Analysis of Variance Table					
**Source**	**df**	**SS**	**MS**	**F-statistic**	**p-value**
Regression	5	1813.06	362.612	11.675	0.017
Residual Error	4	124.24	31.06		
Total	9	1937.299	215.255		

**Fig. 11:**
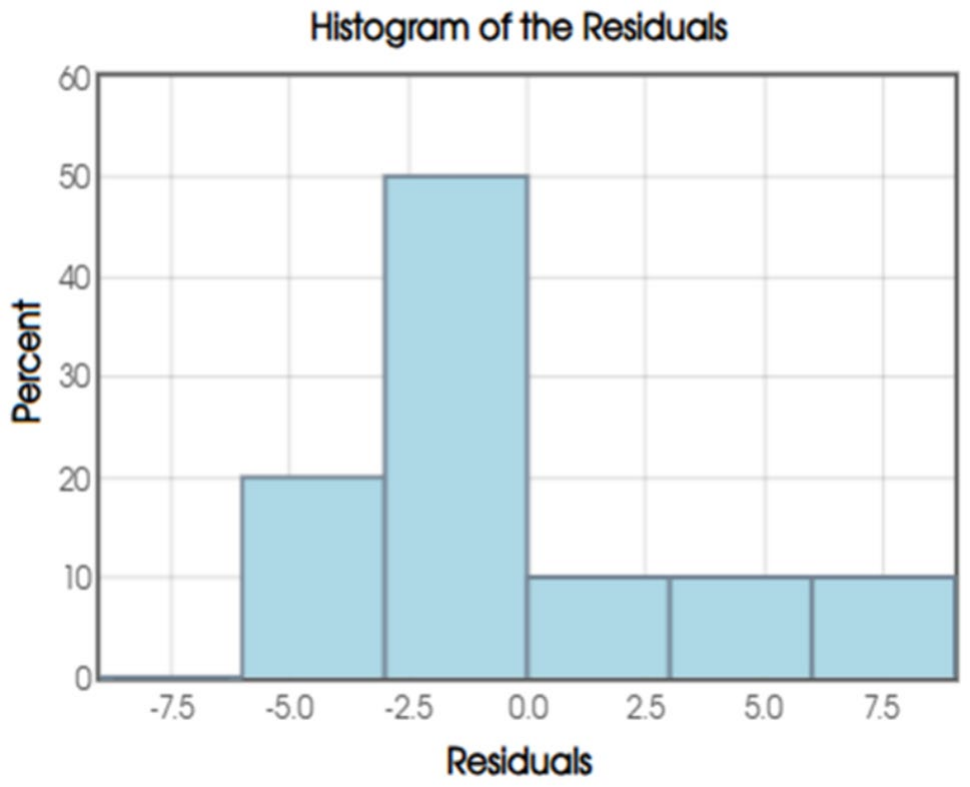
Histogram of the Residuals.

**Fig. 12:**
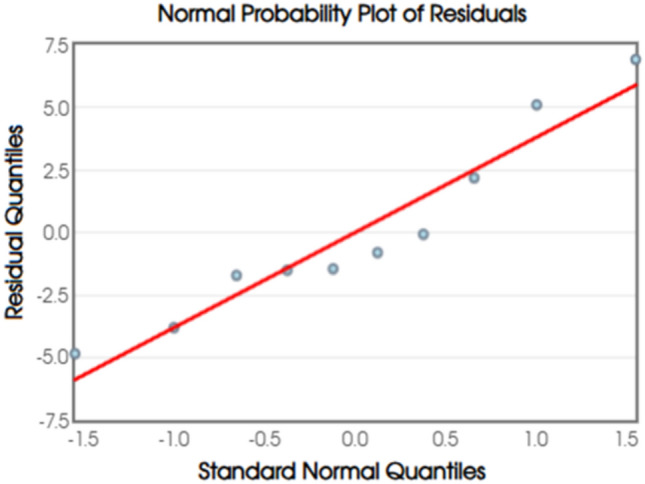
Normal Probability Plot of Residuals.

To assess the assumptions of the BO-MLR model, particularly the normality of residuals, a histogram of the residuals was examined. The distribution of residuals appears to be moderately skewed to the right, with the majority of residual values falling between approximately −3 and 0. Figure 11 shows histogram of the residuals**.** To evaluate the assumption of normally distributed residuals, a normal probability plot was generated. In this plot, the residual quantiles are plotted against the expected quantiles of a standard normal distribution. Ideally, if the residuals follow a normal distribution, the points should align closely with the reference line. Figure 12 shows normal probability plot of residuals.

Table 15 presents the five-number summary of the residuals from the regression model. The minimum and maximum residual values were −4.839 and 6.907, respectively, indicates a moderately wide spread of residuals across both ends. This range suggests that the model exhibits some level of variation in prediction errors, with a tendency for a few larger positive outliers.

**Table 15:** Five Number Summary of Residuals.

Statistic	Value
Minimum (Min)	−4.839
1 st Quartile (Q₁)	−1.713
Median (M)	−1.127
3rd Quartile (Q₃)	2.180
Maximum (Max)	6.907

## Discussion

Form the proposed method, the nitrate and sulphate level are predicted for Chengalpattu area using Sentinel 2 image and deep learning algorithms. Based on the nitrate and sulphate level prediction from Sentinel 2 image, the field visit was conducted and identified the reason for the high level of nitrate and sulphate in those particular locations such as (i) Thiruporur, (ii) Oragadam, (iii) Melmaruvathur, (iv) Anupuram and (v) Maraimalainagar. Interpretation and identification of high level of nitrate and sulphate level regions during different climatic conditions and the reason for the high level are detected and shown in Table 16. During microclimatic conditions such as high temperature and low humidity are analysed. Under high temperature, low humidity is faster organic matter decomposition occurred and releases sulphate and nitrogen compounds, which are then converted to sulphates and nitrates**.** Reduced microbial activity is seen in low temperatures, the microbial processes that drive denitrification slow down, leads to accumulation of nitrate in the soil and eventually in the groundwater. Slower reduction reactions are seen in Sulphate-reducing bacteria (SRB), which convert sulphate to hydrogen sulphide under anaerobic conditions, become less active in cold temperatures, allows sulphate to persist in groundwater. Low temperature preserves nitrate and sulphate in the environment by slowing microbial processes. High humidity facilitates water movement and deposition increases the transport of both nitrates and sulphates into groundwater. These conditions are amplified in urban sprawl areas, where impervious surfaces and waste contribute for more contaminants. Combined, low temperature and high humidity create a hydro chemical setting where nitrate and sulphate concentrations rise is seen in groundwater, particularly in poorly drained or densely populated areas.

**Table 16:**
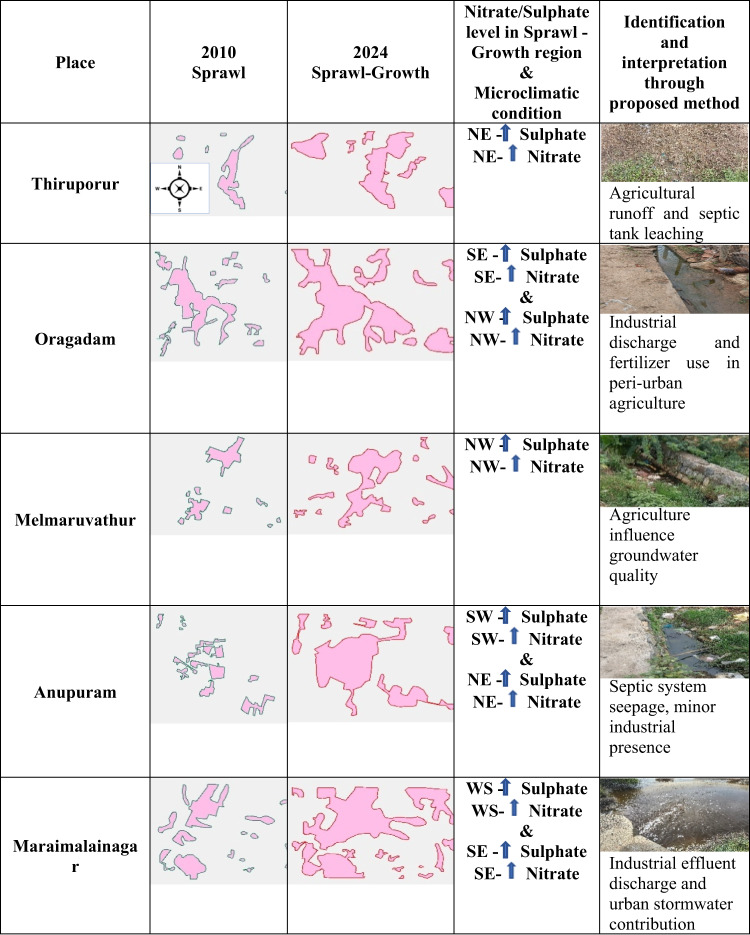
Linking Sprawl Growth and microclimate to Groundwater Degradation.

## Conclusions

The link between the sprawl growth and water contamination levels such as Nitrate and Sulphate are evaluated based on microclimatic condition. Sentinel band is used for Nitrate and Sulphate level prediction. Nitrate and Sulphate indices band is obtained through the ACNN algorithm. From Nitrate and Sulphate indices band combination, the sprawl growth areas are identified in Chengalpattu areas such as (i)Thiruporur (ii)Oragadam (iii)Melmaruvathur (iv)Anupuram (v)Maraimalainagar. For the detected areas the lab values of water contamination is obtained. The contamination water in ground for Nitrate and Sulphate levels depend on certain water parameters. This is the major identification from this proposed study and the area where high Nitrate and Sulphate in groundwater injected is identified. The identification level helps the people and government for proper sewage management system and prevent the contamination levels in groundwater. Further, sprawl grows and its influence on the baseflow of water stream flow in ground need to be studied in future.

## Data Availability

The datasets used during the current study are available from the corresponding author on request.
